# Invisible threats: An investigation of electrical hazards and safety practices among residential electricity consumers

**DOI:** 10.1016/j.heliyon.2024.e34470

**Published:** 2024-07-11

**Authors:** Frank Kulor, Michael W. Apprey, Kafui T. Agbevanu, Gabriel K. Gasper, Judith A. Akorta

**Affiliations:** a Department of Electrical/Electronic Engineering, Ho Technical University, P. O. Box HP 217, Ho, Ghana; b Department of Computer Science, Ho Technical University, P. O. Box HP 217, Ho, Ghana

**Keywords:** Threats, Electrical hazard, Safety awareness, Safety practices, Electricity users

## Abstract

Understanding electrical hazards and implementing safety measures is paramount to protecting lives and property. Therefore, this research investigates electrical hazards in households and safety measures taken by residents in Sokode–Etoe, Ghana. The primary objective is to identify gaps in knowledge regarding electrical hazards among domestic electricity consumers and offer recommendations to enhance safety and mitigate the risks. The data were systematically collected from 200 participants, including both homeowners and tenants, using a structured questionnaire. The results were presented using Likert scale analysis, sample *t*-test, binary logistic regression analysis, involving statistical hypothesis testing of predictor variable coefficients, Importance-Performance Map Analysis (IPMA) and Necessary Condition Analysis (NCA). Participants showed a high awareness of electrical hazards, yet demonstrated a weaker grasp of safety practices, correct emergency procedures, and infrequent testing of wiring systems by homeowners. The predominant electrical accident that emerged was electrical shock. Most homeowners have not engaged certified electrical inspectors for a decade, reflecting uncertainty about the safety protocols in place. Furthermore, respondents expressed a degree of uncertainty regarding the safety measures implemented in their households concerning electricity usage. This study underscores the pressing need to raise awareness and promote safe electrical practices in residential environments. Such an educational initiative could utilize a variety of communication channels, social media influencers, renowned personalities, customised mobile applications and other platforms. This research stands out as the inaugural investigation offering a comprehensive examination of the hazards related to energy consumption and safety precautions in Ghana. It focuses on an often-overlooked demographic of electricity users in Ghana, shedding light on domestic electrical safety issues and the growing hazards.

## 1 Introduction

Electricity stands as the backbone of our contemporary society, illuminating our homes, powering industries, and propelling technological progress [1]. However, amidst the convenience and productivity facilitated by electricity, there exists a concealed realm of potential hazards that pose significant risks to residential electricity consumers. While electricity fuels progress, it also harbours concealed threats within its currents. Failing to grasp and handle these threats with caution can lead to severe consequences, spanning from property damage to life-threatening crises [2].

The fact that electricity cannot be tasted, seen, heard, or scented makes it a "silent killer," even though it is essential to daily living. In essence, it is invisible. If mishandled, it has the potential to cause significant damage to both life and property. Since electricity is invisible, it has long been known to pose a major risk in homes, businesses, educational facilities, and other locations [2].

Frequent fires from electrical faults have become a distressing reality in Ghana, resulting in tragic loss of lives and substantial economic damage annually. Nearly every day, there are reports of fires erupting in various parts of Ghana, instigating fear and alarm among the population [3]. According to data from the Ghana National Fire Service (GNFS) for the year 2009, there were 218 recorded instances of electrical fires out of a total of 2,584 reported fire incidents as of January 25, 2010 [4]. Furthermore, out of a total of 59,933 fire incidents recorded between 2013 and 2022, 23,394 were categorized as domestic fires, constituting 39 % of the total. This statistic underscores the pressing need for enhanced electrical safety education and measures within the community [5]. This alarming trend can be attributed, in part, to a deficiency in knowledge and awareness regarding fundamental electrical safety practices [6].

Again, electric shocks can occur when there are faults or inadequacies in electrical installations. These faults may arise due to various reasons, including incorrect wiring, damaged insulation, or poorly maintained electrical components. Such issues are often exacerbated by a lack of awareness about essential electrical safety practices, which can put individuals at risk. Within residential spaces, where families seek solace, safety, and convenience, these dangers lie in wait, often unnoticed. Fires initiated by faulty wiring, shocks from exposed conductors, and even fatal electrocutions are grim realities stemming from the underestimation of electrical hazards. It is now of paramount importance to shine a light on these lurking dangers and to cultivate a culture of safety awareness among those who utilize residential electricity [7]. Again, a residual current device (RCD) serves as a disconnection device, offering vital safety measures in the event of any unintended contact with a live conductor. An RCD is a safety device that instantly disconnects electrical circuits when it senses an imbalance in the electrical current, which is usually produced by a leakage of current to the earth. They are typically installed within the consumer unit, also known as the fusebox, and have the capability to offer protection for either individual circuits or clusters of circuits. An RCD's threshold, or sensitivity, is often configured to detect very minor imbalances in current flow, usually around 30 mA, for personal safety in domestic installations. When the current imbalance surpasses this threshold, the RCD quickly disconnects the circuit, reducing the risk of electric shock or fire. RCDs are often positioned at the origin of a circuit or integrated into socket outlets to offer localised protection. A highly sensitive RCD can potentially save a person from a life-threatening electric shock [8].

Therefore, every electrical setup necessitates circuit protection to avert potential hazards. Circuit protection serves to promptly halt the flow of current in cases of an overload, ground fault, or short circuit within the wiring system. Electrical grounding entails intentionally establishing a connection with the earth through a low-resistance path [9]. This practice curtails the accumulation of voltage that might lead to an installation mishap. It's important to note that grounding primarily serves as an additional measure against electric shock even though it does not offer an absolute assurance against injury or fatality [2].

Hence, this in-depth investigation aims to uncover a range of electrical hazards and safety practices that often go unnoticed within our homes employing a community-based participatory research framework. This endeavour involves partnering with local communities to undertake an investigation into electrical hazards and safety practices, wherein community members are actively involved in identifying specific safety issues within their homes. This collaborative approach aims to cultivate a sense of ownership and empowerment among community stakeholders towards safety initiatives.

By examining the knowledge, attitudes, and behaviours of residential electricity users, this research aims to address the following critical questions:

1. What are the most common electrical hazards encountered by residential electricity users?
2. To what extent are residential electricity users aware of these hazards, and what is their level of knowledge regarding electrical safety practices?
3. What is the relationship between overall safety in the use of electricity and the awareness of electrical hazards, electrical safety practices, and proper safety procedures?
4. What measures can be implemented to improve awareness and adherence to safety practices among residential electricity users, thereby reducing the incidence of electrical hazards and associated risks?

By addressing these research questions, this study seeks to aid the creation of targeted interventions and educational programs designed to encourage safer electrical practices among residential electricity users. Ultimately, the goal is to enhance safety awareness and prevent avoidable accidents and injuries related to electricity use in homes.

This paper contributes originality by delving into a comprehensive examination of electrical hazards and safety practices within residential settings. By focusing on often overlooked aspects of electrical safety, the current study fills gaps in the existing literature on electrical issues among residential users in Ghana, offering a new perspective on individuals' perceptions of electricity usage and providing valuable insights into the complexities of electrical safety within residential environments. This not only informs the government and stakeholders about the increasing threat, but also lays the groundwork for heightened awareness campaigns, effective policy implementation, and educational initiatives in addressing the multifaceted challenges of electrical safety in residential settings. Through education and awareness, we strive to transform passive electricity consumers into vigilant protectors of their homes.

## 2 Literature review

### 2.1 Electrical system standards and safety measures in Ghana

In Ghana, the electrical system runs at a standard voltage of 220–240 V, and a frequency of 50 Hz. This voltage level is comparable with international standards and allows for compatibility with electrical appliances and equipment typically used in homes and workplaces. Household electrical voltages normally range between 220 and 240 V, providing enough power for a variety of electrical gadgets and appliances [10]. Circuit protection methods are used to prevent potential hazards like electrical fires or shocks caused by overcurrents. These preventive measures are often in the form of circuit breakers or fuses, which automatically halt the flow of electricity when abnormal current levels are detected [7].

### 2.2 Implementation of the Ghana electrical wiring regulation

Developing safety policies and procedures is crucial for reducing the risk of injuries and deaths. Therefore, implementing comprehensive policies and regulations is essential to ensure the safe use of electricity in the country. In response to a series of electrical fires, the Ghana Electrical Wiring Regulation 2011, L.I.2008, was enacted by Parliament on Friday, February 24, 2012 [11]. The primary goal of these regulations is to ensure that electrical wiring and installation work is conducted only by properly qualified and certified professionals. These professionals must adhere to well-defined standards that safeguard people, assets, and animals. According to these regulations, only Certified Electrical Wiring Professionals (CEWPs) who meet the required qualifications are legally permitted to perform domestic wiring [12]. Additionally, the regulations mandate mandatory inspection and testing of installations before they are put into service to verify compliance with safety standards.

### 2.3 Regulatory management of Ghana's electrical infrastructure

The Energy Commission, Ghana Standard Authority (GSA), and the Electricity Company of Ghana (ECG) share responsibility for the regulatory management of Ghana's electrical infrastructure. These organizations establish and enforce standards and regulations to ensure the safe installation, maintenance, and operation of electrical systems across the country. Their mission is to protect the integrity of the electrical grid and promote public safety. Furthermore, the Public Utilities Regulatory Commission (PURC) is responsible for overseeing the price, value, and accessibility of utility services such as electricity. PURC aspires to balance the interests of consumers and utility providers by regulating price structures and monitoring service delivery, all while promoting a sustainable and efficient electrical supply network. These regulatory agencies work together to ensure the dependability, safety, and efficiency of Ghana's electrical infrastructure, thereby promoting economic development and improving the quality of life for individuals across the country [13].

### 2.4 Compliance and enforcement

The regulations mandate that the Energy Commission publish guidelines for certifying electricians within six (6) months of the regulations becoming effective. These guidelines encompass the certification process, application procedures, exemptions, transitional provisions, and enforcement mechanisms. Recently, an additional requirement has been introduced to the certification process, stipulating that Certified Electrical Wiring Inspectors (CEWIs) must inspect the work performed by Certified Electrical Wiring Professionals (CEWPs) before granting approval [14].

Overall, the implementation of these comprehensive policies and regulations is essential to ensure the safe use of electricity in Ghana. By establishing stringent standards and certification processes, the country aims to enhance public safety, prevent electrical hazards, and promote the reliable operation of its electrical infrastructure.

### 2.5 Current landscape of research on electrical hazards and safety measures

Scholarly research on the safety standards of residential buildings and the level of awareness regarding electrical installations has been conspicuously lacking in recent years. This lack of academic attention has resulted in a discernible gap in available data within this particular domain, hindering comprehensive understanding and effective intervention strategies. The existing body of research concerning this subject matter, as delineated in Table 1, is relatively limited but provides valuable insights into various aspects of electrical safety within residential settings. These studies offer a foundation upon which further investigation and analysis can be built, aiming to address the identified research gap and enhance our understanding of the complex interplay between residential infrastructure, electrical hazards, and public awareness.

**Table 1:** Studies related to electrical hazards and safety measures.

S/No.	Reference/	Year of pub.	Research aim	Findings
1.	[15]	2012	Evaluated the effectiveness of Home Safety Assessment (HSA) intervention in preventing fire-related deaths and injuries.	There were significant reductions in accidental dwelling fire rates and related injuries, though the proportion of fires contained to the room of origin remained unchanged.
2.	[16]	2013	Evaluated the state of electrical setups in residential dwellings and apartment complexes situated in the northern region of Jordan.	The primary finding of this study suggests that the majority of residences lack essential safety precautions as a result of inadequate implementation and amateur electrical wiring, compounded by the absence of required oversight.
3.	[2]	2014	Analyzed the degree of electrical dangers and safety measures knowledge among Minna metropolitan power customers.	The consumers have an understanding of the dangers associated with improper electrical installation and damaged electrical appliances and equipment but lack awareness regarding the risks posed by ungrounded circuits, equipment, and coiled extension leads.
4.	[17]	2017	Assessed the attitudes associated with electrical safety in Saudi Arabia's Hail region.	Utilizing a scale akin to the one used for calculating university student GPAs, they determined an electrical safety level (ESL) of 0.76 on a 4-point scale. This low score signifies a deficient electrical safety culture in the Hail region.
5.	[18]	2017	Examined the awareness of electrical safety among the millennial population in Chennai, Tamil Nadu.	The findings highlight that sockets pose a potential risk and require measures to ensure the safety of individuals, particularly children. It was crucial to regularly inspect and replace these to prevent hazards, overloads, and short circuits.
6.	[1]	2019	Examined the degree of adherence to electrical safety protocols among faculty and undergraduates within the EE department at Bayero University, Nigeria.	The results unveiled the typical triggers of electrical mishaps in workshops and laboratories, highlighting a lack of strict adherence to electrical safety protocols by both staff and students.
7.	[19]	2019	Evaluated the extent of electrical risks and awareness regarding safety measures among consumers of electricity in Sokoto State, Nigeria.	It was revealed that energy users are conscious of improper electrical installations, which consequently lead to the damage of electrical and electronic devices and equipment.
8.	[8]	2020	Discussed the significant progress in electrical safety, encompassing various industrial, commercial, and residential aspects.	The RCD serves as a safeguarding mechanism that swiftly identifies electrical faults and automatically cuts off the electricity supply.
9.	[20]	2021	A secure protocol for conducting routine inspections of residential electrical installations in Bayelsa State was introduced.	Consistent examination and evaluation of electrical setups in residential properties could notably diminish the frequency of electrical fires in buildings throughout Bayelsa State. This practice would consequently guarantee the safety of both the structure and its inhabitants.
10.	[21]	2022	Outlined the perspective on occupational safety and health concerning electrical hazards within hospital environments.	The study concluded that electrical dangers pose a notable risk within hospital settings, necessitating ongoing evaluation and enhancement efforts.
11.	[7]	2023	Assessed the safety practices of students in using power extension cords in their accommodations.	The results showed that approximately 52 % of the participants lacked knowledge about the ratings, as well as the consequences of overburdening an extension cord.
12.	[22]	2024	Assessed the extent of safety precaution awareness regarding the use of extension cords in Ho, Ghana.	Participants demonstrated limited awareness of ratings and standard labelling of extension cords provided by testing facilities. Additionally, most overload cords and practice poor safety measures, increasing fire risk.

The existing body of literature in this research area is notably deficient in comprehensive data. This gap is conspicuous, as it hinders a thorough understanding of the extent to which residential electricity consumers are cognizant of potential risks and equipped with the necessary knowledge to implement safety measures effectively. This scarcity of data points to a critical area within electrical safety that requires further investigation and exploration.

## 3 Materials and methods

### 3.1 Study area

**Fig. 1:**
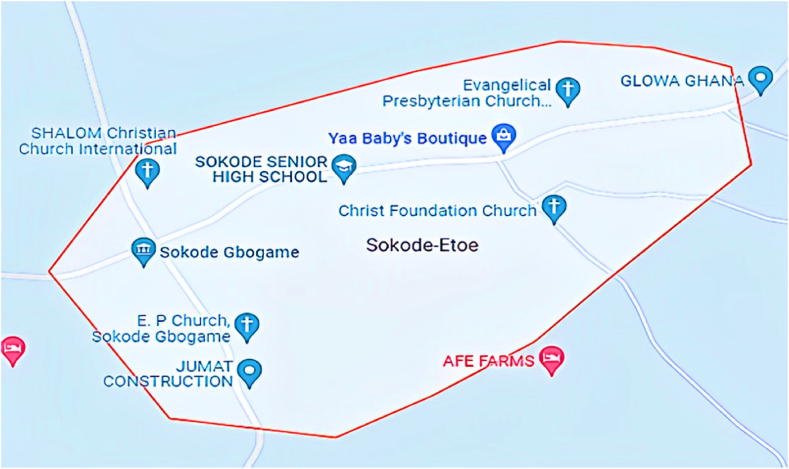
Map of Sokode – Etoe [24].

The population for the collection of data is the electricity users and residential buildings in Sokode-Etoe in the Ho Municipality of the Volta Region of Ghana. Sokode-Etoe is a place with a significant population in Ho Municipality, Ghana [23]. Sokode-Etoe is situated northeast of Sokode Gbogame. The town depicted in Fig. 1 is situated between latitudes 6.57408° or 6° 34′ 27″ north, and longitudes 0.41333° or 0° 24′ 48″ east [24].

### 3.2 Sample size estimation

A purposive sampling strategy was employed to select a total of 200 participants. Researchers utilized the normal approximation to the hypergeometric distribution formula, as outlined in Eq. (1), originally developed by Ref. [25] and employed by Refs. [26,27] for estimation in various research endeavours. This computation relied on specific assumptions regarding the selected electricity users and residential buildings (N) of 245, both the percentage of success (p) and failure (q) rates were set at 50 %, an error margin (E) of 3 %, and a standard score value (Z) of 1.96 and C·I. - 95 %. Following the calculation, as demonstrated in Eq. (2), the determined required sample size (n) was set at 200.

(1) $$n=\frac{NZ^{2}\text{pq}}{\left(E^{2}\left(N-1\right)+Z^{2}\text{pq}\right)}$$

Using the formula provided above, the sample size was calculated as:

(2) $$n=\frac{\left(245\right)*{\left(1.96\right)}^{2}*\left(0.5\right)*\left(0.5\right)}{\left({\left(0.03\right)}^{2}\left(245-1\right)+{\left(1.96\right)}^{2}*\left(0.5\right)*\left(0.5\right)\right)}=200$$

**Fig. 2:**
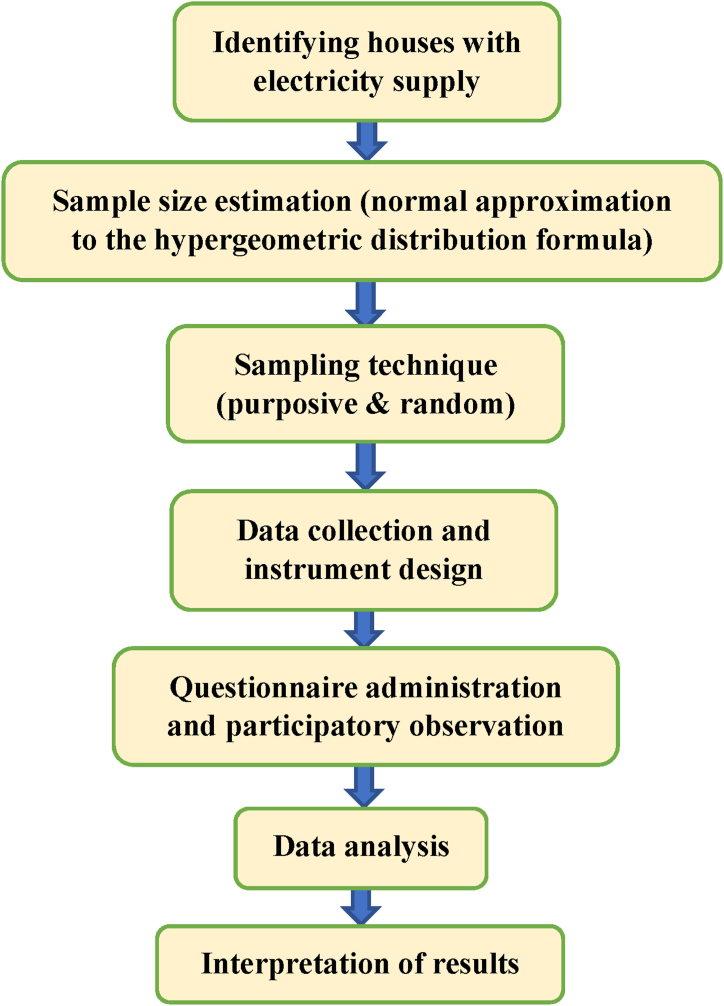
Flow chart of the research method.

Fig. 2 displays a work flow chart outlining the steps for evaluating the electrical hazards and safety practices among residential electricity users.

### 3.3 Data collection and instrument design

In this research, data were collected from respondents through face-to-face interviews using a structured questionnaire in January and March 2022. This guarantees that all participants receive identical questions presented consistently, thereby minimizing variability and bolstering the reliability of the data, a practice commonly adopted by numerous researchers [22,27,28]. Respondents were presented with a set of questions and asked to select their responses. The participants for the questionnaire were selected using purposive sampling. This method was selected to ensure that heads of households with electricity supply were adequately represented in the study. In instances where multiple eligible respondents were present in a single designated residence, a lottery method was employed to select a respondent. Visiting systematically chosen residences with electricity, data collectors conducted interviews with either the heads of households or any household member aged 18 years or older [29].

Additionally, a random sampling method was employed to choose 50 homeowners as a focus group (FG), aiming to inspect their electrical installations at their premises and gather detailed qualitative insights and opinions.

The survey consisted of five sections, primarily comprising close-ended questions. The first part focused on gathering basic demographic data. The subsequent section evaluated participants' general awareness of electrical hazards, knowledge of appropriate safety protocols during emergencies, and any previous involvement in electrical accidents. The third section delved into respondents' awareness of safety practices associated with the use of electrical appliances and equipment. The fourth section delved into homeowners' perspectives on the general inspection and testing of electrical installations in their premises. Finally, the concluding section explored participants' opinions on the level of safety protection within residential buildings. To ensure clarity and understanding, the questions were clarified for respondents who encountered difficulty in answering the questionnaire.

### 3.4 Data processing and statistical techniques

A total of two hundred (200) participants took part in this study. The responses from the questionnaires were carefully examined for any errors, inconsistencies, or contradictions. Analyzing responses from the questionnaire to detect errors, inconsistencies, or contradictions is crucial in upholding the accuracy, integrity, and validity of the research. Identifying and resolving such issues will elevate the quality of the research findings and guarantee that conclusions are grounded in dependable evidence.

Both Statistical Package for Social Sciences (SPSS) and SmartPLS software were used for data processing. The statistical techniques employed in this study encompassed binary logistic regression, Likert scale analysis, one-sample *t*-test, descriptive statistics, IPMA, and NCA. The statistical technique of binary logistic regression examines the relationship between a binary dependent variable, typically represented as 0 or 1, and one or more predictor variables [30]. The examination of the influence of several factors on the chance of an event occurring is particularly advantageous. The process of Likert scale analysis involves evaluating the responses to survey inquiries by employing a Likert scale, wherein participants express their degree of concurrence or dissent towards a set of claims. The use of this approach enables researchers to quantitatively assess subjective opinions or attitudes and examine patterns inherent in the collected data. The one-sample *t*-test is a statistical procedure employed to ascertain the presence of a significant difference between the mean of a single sample and a known or hypothesized population mean. The method is frequently employed to evaluate the presence of a statistically significant disparity between the mean of a sample and a predetermined value. The presented statistics offer a succinct and comprehensive summary of the data, facilitating the analysis and understanding of research outcomes [27,30]. Using both IPMA and NCA together provides a thorough analysis by identifying not only which factors are important and how well they are performing (IPMA) but also which factors are essential and cannot be ignored (NCA). The combination allows for a balanced approach to improvement strategies, ensuring that critical necessary conditions are met while also focusing on enhancing important but underperforming areas.

Additionally, the researchers conducted on-site interviews and observations of the electrical installations in the vicinity covered by the study as agreed by the participant. They closely examined the installations and inspected equipment when necessary. These observations aided in gathering supplementary information and supported the researchers in forming accurate and pertinent arguments for discussion.

## 4 Results

The main objective of the research was to assess the awareness regarding electrical safety among electricity users in Sokode–Etoe, Ghana. The information collected from the participants via the questionnaire underwent thorough analysis and was then presented as the findings of the study.

### 4.1 Bio-data of respondents

Table 2 provides an overview of the respondents' demographics. It reveals that 60 % of the respondents were male, while females accounted for 40 %. This indicates a male predominance in the township, which is significant for the study since, in many societies, males are generally responsible for matters concerning the family, including the safety of the house.

**Table 2:** Demographic characteristics of participants (n = 200).

Demographic Characteristic	Category	Frequency (n)	Percentage (%)
Gender	Male	120	60.0
	Female	80	40.0
	**Total**	**200**	**100.0**
Age (Years)	20–25	45	22.5
	26–45	63	31.5
	46–60	42	21.0
	Above 60	50	25.0
	**Total**	**200**	**100.0**
Educational Status	None	12	6.0
	JHS	27	13.5
	SHS	100	50.0
	Tertiary	61	30.5
	**Total**	**200**	**100.0**
House Status	Owner	115	57.5
	Tenant	85	42.5
	**Total**	**200**	**100.0**

Moreover, the data reveals that 21.5 % of the respondents were aged between 20 and 25, 31.5 % were between 26 and 45 years old, 21 % fell between 46 and 60 years old, and 25 % were above 60 years of age. This indicates that all age groups in the township were included in the survey, making the sample representative of the entire population.

The findings also indicate that 6 % of the respondents had received no formal education, while 13.5 % were Junior High School (JHS) graduates. A significant portion, accounting for 50 % of the participants, had completed Senior High School (SHS), and 30.5 % were tertiary school graduates. This outcome suggests that most respondents possessed at least a second-cycle level of education.

Lastly, the housing status of the respondents was examined. As shown in Table 2, approximately 57.5 % of the respondents were homeowners, while 42.5 % were tenants. Generally, homeowners tend to have greater awareness regarding safety-related matters [17].

### 4.2 Awareness of electrical hazards and proper safety procedures

#### 4.2.1 Level of electrical hazard awareness

Table 3 describes the outcome of the general awareness of some potential hazards associated with the use of electrical appliances and equipment.

**Table 3:** Awareness of electrical hazards.

S/No.	Item	Aware (%)	Not Aware (%)	Mean score
1.	Coiling extension leads on the drum.	30.0	70.0	1.70
2.	Covering ventilation holes in electrical equipment.	73.5	26.5	1.26
3.	Use of high-wattage lamp.	23.5	76.5	1.76
4.	Removing the plug from an electrical socket by tugging on the cord.	78.0	22.0	1.22
5.	Overloading of extension cords.	19.0	81.0	1.81
6.	Use of damaged outlets and switches.	82.0	18.0	1.18
7.	Use of systems with exposed wires.	80.5	19.5	1.20
	**Average mean score**			**1.45**

Utilizing Likert scale evaluation as a statistical method, let;

H_0_ = general awareness of potential hazards is high.

H_1_ = general awareness of potential hazards is low.

SPSS code; Aware = 1 and Not aware = 2.

Critical value = 1.5; Accept if the average mean score is above the critical value and reject if ii is below.

With an average mean score of 1.45, as computed in Table 3, falling below the critical value of 1.5 necessitates rejecting the null hypothesis. This suggests that the general awareness of potential hazards linked to the usage of electrical appliances and equipment among the respondents is perceived as high.

#### 4.2.2 Awareness of proper safety procedures

Table 4 depicts the result of the general awareness of the proper procedures to follow in the event of an electrical emergency (e.g., short circuit, electrical fire) using binary logistic regression analysis. The respondents' biodata were utilized as independent variables to predict the observed outcomes in this analysis.

**Table 4:** Awareness of proper safety procedures.

Test	Step 1	Wald	Sig.	Exp(B) (OR)	EXP. (B) of 95.0 % C·I.	
					Lower	Upper
General awareness of the proper procedures to follow in the event of an electrical emergency.	Gender	7.469	0.006	2.543	1.302	4.968
	Age	2.334	0.127	1.271	0.934	1.728
	Education	2.041	0.153	1.328	0.900	1.961
	Status	0.885	0.347	1.384	0.703	2.723

Test of model coefficient Chi^2^ = 11.388; df = 4, p = 0.023.

Hosmer and Lemeshow test: Chi^2^ = 5.553; df = 8; p = 0.697.

Nagelkerke R^2^: 8.2 %.

Classification Accuracy: 75 %.

Predictor(s) entered on step 1: Biodata of respondents.

The results from the Hosmer and Lemeshow goodness of fit test indicate that the data fits the model well, as the p-value is nonsignificant at the 5 % level (Chi^2^ = 5.553; *p-value* = 0.697 > 0.05). A higher likelihood of being aware of the correct procedures to follow during an electrical emergency was observed among males, indicating a gender-related difference in awareness. The odds ratio (OR) and the exponential coefficient EXP (B) for the predictor, calculated with a 95 % confidence interval (CI) as shown in Table 4, were Wald = 7.469 (Exp(B) = 2.543; 95 % CI: 1.302–4.968), with a p-value of 0.006 (<0.05). This indicates that gender is a significant factor at the 5 % level. The predictors age, education and house status with their *p-values* greater than 0.05 are not significant predictors of general awareness at the 5 % level as indicated in Table 4. Based on the Nagelkerke R^2^ test model, the selected predictors accounted for 8.2 % of the variance in the awareness level, indicating limited predictive power. The results indicated that 149 participants (74.5 %) were unaware of the proper safety procedures to follow during an electrical emergency, whereas 51 participants (25.5 %) were aware of these procedures. This led to an overall prediction accuracy of 75 %.

#### 4.2.3 Involvement in electrical accidents

**Fig. 3:**
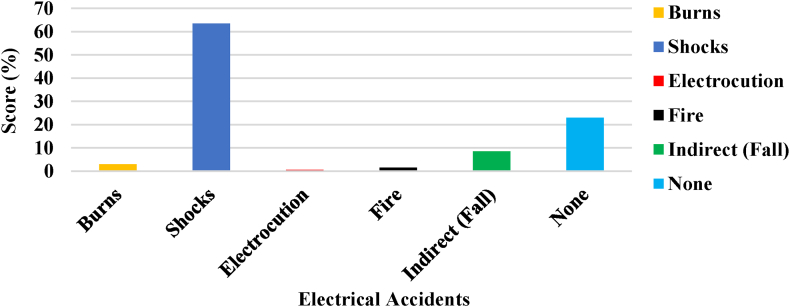
Involvement in electrical accidents.

Fig. 3 illustrates the evaluation of the incidents and injuries related to electricity encountered by the participants.

It indicates that 63.5 % of the respondents encountered electrical shock, while 8.5 % faced an indirect hazard (trip and fall). Additionally, 3 % of the participants suffered from electrical burns, whereas 0.5 % and 1.5 % experienced electrocution and minor fire incidents, respectively. Notably, about 23 % of the respondents reported no experience of any of the aforementioned incidents. This suggests that about 77 % of the respondents have suffered from electrical accidents at home.

### 4.3 Awareness of electrical safety practices

Table 5 describes the outcome of the awareness of some safety practices adopted during and after the use of electrical appliances and equipment.

**Table 5:** Awareness of safety practices.

S/No.	Item	Aware (%)	Not Aware (%)	Mean score
1.	The extension cord is fully uncoiled from the drum.	26.0	74.0	1.74
2.	Unplug unused devices.	75.5	24.5	1.24
3.	Avoid plugging the heater on an extension cord.	17.5	82.5	1.82
4.	Avoid touching power equipment with a wet body.	89.0	11.0	1.11
5.	Conduct regular inspections.	22.5	77.5	1.78
6.	Avoid overloading circuits.	19.5	80.5	1.80
	**Average mean score**			**1.58**

Utilizing Likert scale evaluation as a statistical method, let;

H_0_ = general awareness of safety practices is high.

H_1_ = general awareness of safety practices is low.

SPSS code; Aware = 1 and Not aware = 2.

Critical value = 1.5; Accept if the average mean score is above the critical value and reject if ii is below.

With an average mean score of 1.58, as computed in Table 5, falling above the critical value of 1.5, it is necessary to accept the null hypothesis. This implies that there exists a relatively limited awareness regarding safety practices during the use of electrical appliances and equipment.

### 4.4 Inspection and testing of electrical installation

#### 4.4.1 Knowledge of inspection and testing methods

**Fig. 4:**
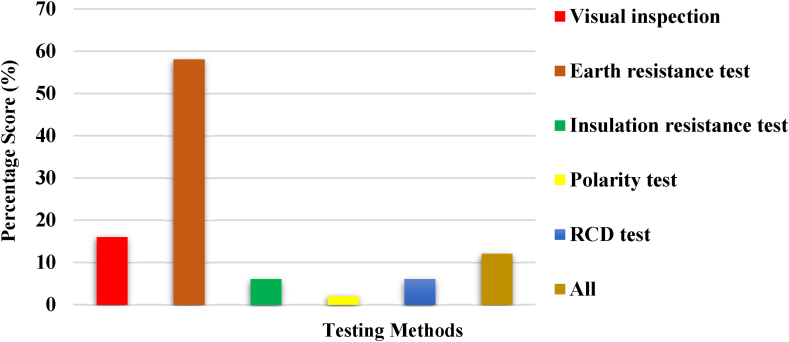
Knowledge of inspection and testing methods.

The focus group's familiarity with diverse electrical testing methods employed during the inspection and testing of electrical installations was evaluated and displayed in Fig. 4. In all, the majority of the respondents, representing 58 %, knew about earth resistance tests; 18 % knew about visual inspection; 6 % knew about insulation resistance and RCD tests; 2 % knew about polarity tests; and 12 % had knowledge of all. This suggests that respondents have some knowledge of electrical installation tests to ensure safety in their residences.

#### 4.4.2 Inspection and testing by certified electrical wiring professionals and inspectors

**Fig. 5:**
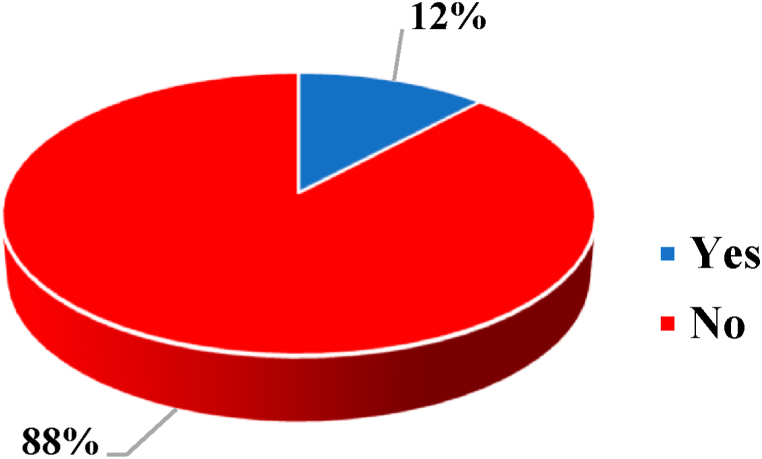
Inspection and testing of electrical installation.

The assessment inquired about the inspection and testing of electrical installations by a certified electrician are displayed in Fig. 5. A focus group comprising solely 50 homeowners with buildings in use for over ten (10) years was convened to gather their perspectives on whether they had ever inspected and tested their electrical installation within the past 10 years. Based on the survey results, it was found that 88 % of respondents stated they hadn't enlisted the services of a Certified Electrical Wiring Professional (CEWP) and Certified Electrical Wiring Inspector (CEWI) for inspection and testing in the past 10 years, while only 12 % had sought their services.

### 4.5 Overall level of safety in the use of electricity

In this section, we unveil the results regarding the overall safety awareness of the respondents and their families in utilizing electricity. This assessment was conducted using a 5-point Likert scale analysis. The analysis was done as a two-tailed test with alpha (α) = *0.05*.

The analysis of the data presented in Table 6 reveals that, on average, respondents are uncertain about the overall level of safety in the use of electricity, as indicated by a mean score of (3.04 ± 0.91) on a Likert scale ranging from 1 to 5. The *t*-test results, with a t-value of −30.400 and a significance level of 0.000, signify a significant deviation from the test value of 5, suggesting that respondents' perception of safety is notably lower than strongly agreeing with safety measures. The negative mean difference of −1.955 and the 95 % confidence interval for the difference −1.83 to −2.08, both excluding zero, further emphasize the significant difference from the test value. Additionally, the Shapiro-Wilk test results demonstrate that the data deviates from a normal distribution (p < 0.05).

**Table 6:** Overall level of safety in the use of electricity.

Test	Test Value = 5					
	t	df	Sig. (2-tailed)	Mean Diff.	95 % C·I of the Difference	
					Lower	Upper
Overall level of safety in the use of electricity.	−30.400	199	0.000	−1.955	−2.08	−1.83
*N - 200* *Mean - 3.04* *Std. Deviation - 0.909* *Std. Error Mean - 0.064* *Shapiro-Wilk's Test - (p < 0.05).* *Critical Value - 3.0*						

Mean (1 = Strongly Disagree; 2 = Disagree; 3 = Uncertain; 4 = Agree; 5 = Strongly Agree).

As a result, the study concludes that there is an indeterminate level of overall safety in the utilization of electricity, thereby supporting the null hypothesis as the mean value (M = 3.04) obtained exceeds the critical value of 3.0.

### 4.6 PLS-SEM analysis

Two advanced analyses, namely, IPMA and NCA were conducted to elucidate both the importance and ceiling effects of awareness of electrical hazards, awareness of electrical safety practices, and awareness of proper safety procedures on overall safety in the use of electricity.

#### 4.6.1 Importance-performance map analysis (IPMA)

**Fig. 6:**
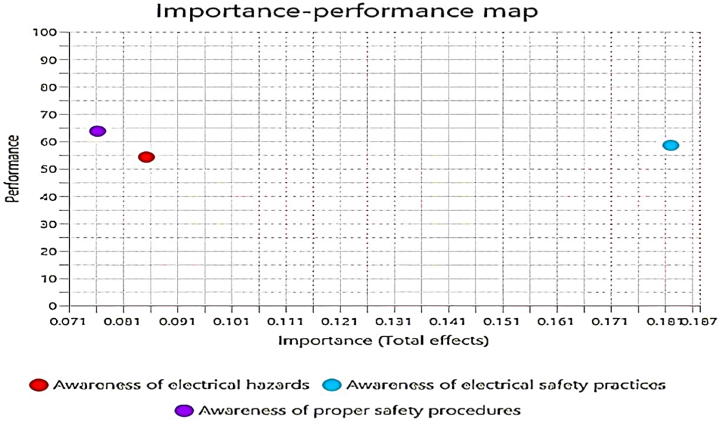
IPMA findings.

The IPMA results in Table 7 and Fig. 6 underscore that awareness of electrical safety practices is the most crucial factor influencing overall safety in the use of electricity, with the highest importance (0.182) and moderate performance (58.565). Awareness of electrical hazards, although moderately important (0.085), has lower performance (54.281), indicating a need for improvement. Conversely, awareness of proper safety procedures shows the highest performance (63.750) but the lowest importance (0.076). Thus, prioritizing the improvement of awareness of electrical safety practices will yield the most significant impact on enhancing overall safety in the use of electricity. Simultaneously, there is a need to improve the performance of awareness regarding electrical hazards, given its substantial importance. Although the awareness of proper safety procedures is performing well, maintaining its high performance remains necessary for comprehensive safety management.

**Table 7:** IPMA statistics.

Predictors	Total effects	Performance
Awareness of electrical hazards	0.085	54.281
Awareness of electrical safety practices	0.182	58.565
Awareness of proper safety procedures	0.076	63.750

#### 4.6.2 Necessary condition analysis (NCA)

The NCA ceiling line charts provide valuable insights into the relationship between awareness of electrical safety and overall safety in the use of electricity. The analysis focused on three specific predictors: awareness of electrical hazards, awareness of electrical safety practices, and awareness of proper safety procedures. Each chart illustrates how these awareness factors influence the overall safety of electrical usage.

**Fig. 7:**
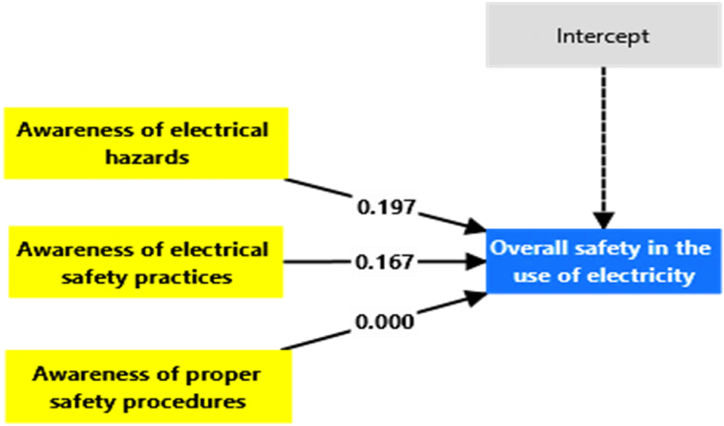
Graphical NCA results.

**Fig. 8:**
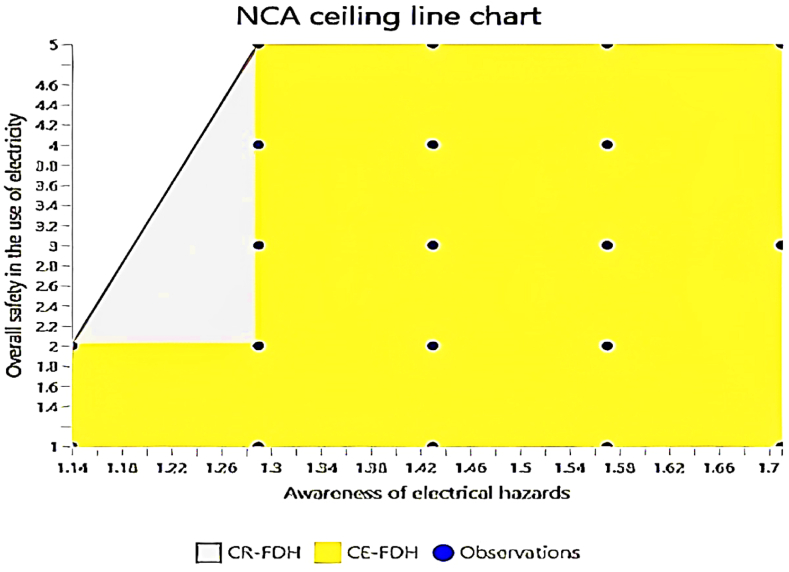
NCA plot (Awareness of electrical hazards and overall safety in the use of electricity).

**Fig. 9:**
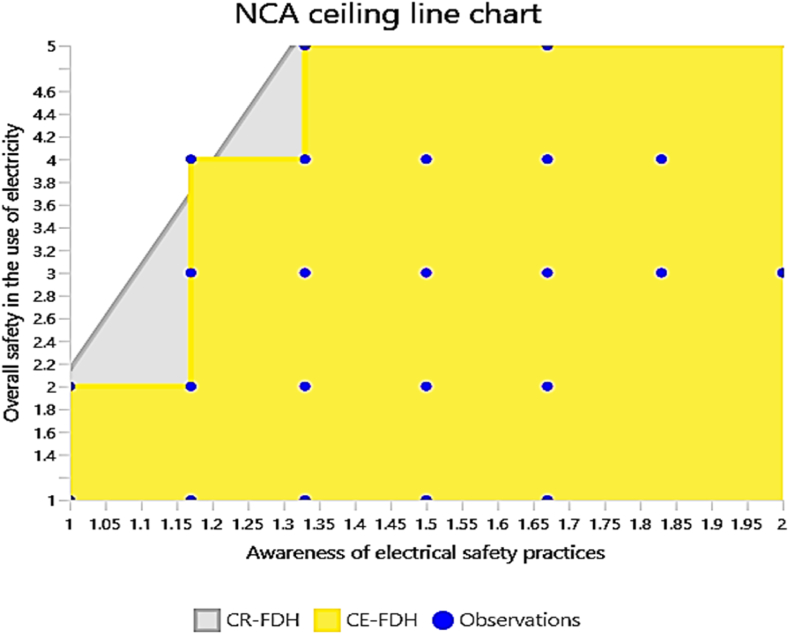
NCA plot (Awareness of electrical safety practices and overall safety in the use of electricity).

**Fig. 10:**
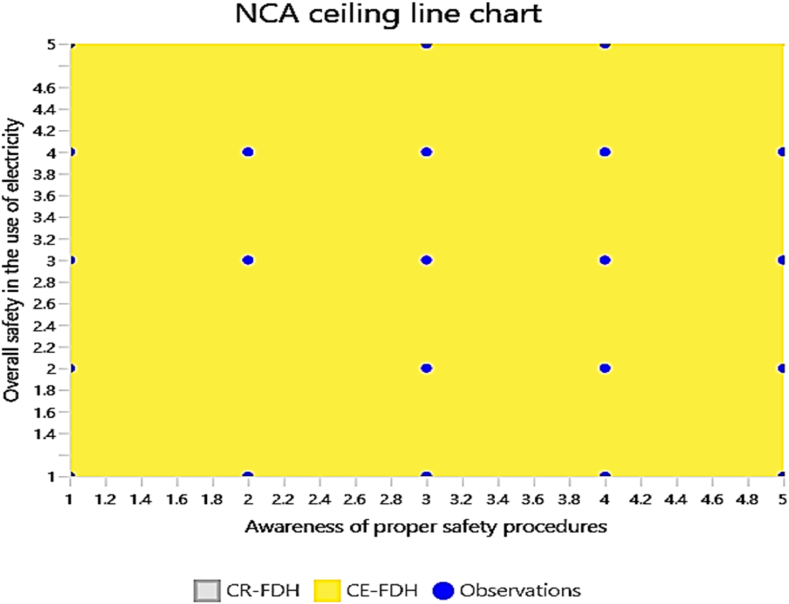
NCA plot (Awareness of proper safety procedures and overall safety in the use of electricity).

The NCA results as summarised in Table 8 and visualised in Fig. 7, Fig. 8, Fig. 9, Fig. 10, validate the criticality of these factors by demonstrating their ceiling effects on overall safety. Awareness of electrical hazards exhibits the highest ceiling effect (CE-FDH: 0.197, CR-FDH: 0.099), indicating its substantial role in setting the upper limits of achievable safety. Similarly, awareness of electrical safety practices shows notable ceiling effects (CE-FDH: 0.167, CR-FDH: 0.112), reinforcing its importance highlighted in the IPMA. However, awareness of proper safety procedures does not present any ceiling effect (CE-FDH: 0.000, CR-FDH: 0.000), suggesting that while this factor performs well, it does not constrain the maximum achievable safety levels. Fig. 7 provides an overarching graphical summary of the NCA results.

**Table 8:** NCA statistics.

Predictors	CE-FDH	CR-FDH
Awareness of electrical hazards	0.197	0.099
Awareness of electrical safety practices	0.167	0.112
Awareness of proper safety procedures	0.000	0.000

Fig. 8 specifically plots the relationship between awareness of electrical hazards and overall safety in the use of electricity, clearly showing the significant ceiling effect this predictor has. Fig. 9 illustrates the NCA plot for awareness of electrical safety practices, further confirming its notable influence on overall safety. Lastly, Fig. 10 demonstrates the NCA plot for awareness of proper safety procedures, reinforcing the absence of a ceiling effect for this predictor. This chart highlights a similar trend, where higher awareness is associated with improved safety outcomes. Notably, there is a broader spread of data points indicating a more substantial correlation between these variables.

Overall, these results highlight the necessity of focusing on enhancing awareness of electrical hazards and safety practices to achieve substantial improvements in electrical safety. These factors exhibit significant ceiling effects, indicating their critical role in defining the upper limits of safety. In contrast, awareness of proper safety procedures, while performing well, does not impose a ceiling effect, suggesting that other factors are more pivotal in elevating safety standards to their highest potential. Therefore, targeted interventions to enhance awareness of electrical hazards and safety practices are essential for substantial improvements in electrical safety.

## 5 Discussion

### 5.1 Summary of findings

This research evaluated the awareness level regarding electrical hazards and safety protocols among electricity users in Sokode–Etoe, Ghana. It is the first of its kind in the municipality and the region as a whole.

The study revealed that the participants possessed some knowledge level regarding hazards in the use of electricity. This is in agreement with [2], where participants were aware of some hazards in the use of electricity in Minna Metropolis of Niger State, Nigeria. Respondents were equally unaware of some important hazards such as the overloading of extension cords and the use of high-wattage lamps. An inspection done during this research in some homes of the participants truly revealed this dangerous practice. The respondents lacked awareness regarding extension cord ratings and testing laboratories. The majority of extension cords were not certified by any testing laboratory or endorsed by the Ghana Standard Board. Some respondents even reported using homemade extension cords. In interviews and discussions with participants, they revealed various reasons for their diverse practices in using extension cords:

“The extension cord's ample number of outlets and its ability to cater to our daily need for powering multiple electronic devices, even when they're situated far from a single power source, are highly convenient features. However, we often lack awareness regarding the power and current ratings of the extension cord; our primary focus is on conveniently connecting multiple devices to it. Additionally, homemade extension cords are preferred due to the perception of better durability and the use of sturdier materials compared to some imported ones.”

This corroborates with the research conducted by Refs. [7,31], where the studies revealed that extension cords were overloaded in over 90 % of homes visited. It's important to remember that every extension cable has a specific wattage capacity, influenced by factors like wire thickness and cable length. Using too many appliances at once can surpass this limit, causing potential damage and overheating. In rare cases, this overheating, which is an invisible threat, can lead to flames and harm to the cable's components. To prevent these risks, always ensure you don't overload the extension cable [32,33]. Again, using higher wattage lighting fixtures at home results in increased brightness, higher energy consumption leading to potentially higher bills, potential risk of overheating if fixtures aren't rated for higher wattage, shorter lifespan for bulbs, harsher and potentially glare-inducing lighting, potential mismatch with desired ambience, and increased environmental impact due to greater energy consumption. The effects of poor awareness of electrical hazards extend far beyond the immediate incident. They can have significant and lasting impacts on individuals' well-being, financial stability, and emotional health, as well as on broader community safety [34]. Extension cords should only be employed for temporary needs and in situations where they are truly indispensable. They should never be considered a replacement for the need to have outlets and proper wiring installed where they are needed [7]. This underscores the critical importance of promoting electrical safety education and awareness at both individual and societal levels.

The respondents also expressed that they are unaware of the proper safety procedures to follow in the event of an electrical emergency (e.g., short circuit, electrical fire). The majority of people often struggle to execute proper safety procedures during an electrical emergency due to a combination of factors. These include a lack of formal training and knowledge in electrical safety, heightened stress levels and panic that impede clear thinking, a fear of exacerbating the situation, and an unfamiliarity with electrical systems. Additionally, inadequate preparedness, physical barriers, overestimation of personal capabilities, language barriers in multicultural settings, and a lack of regular practice in emergency response can all contribute to this challenge [35]. Remarkably, homes were lacking fire extinguishers. The GNFS has urged Ghanaians to enforce the mandatory presence of fire extinguishers in households. This proactive measure ensures a rapid response in case of fire emergencies, ultimately safeguarding lives and property. The GNFS has expressed apprehension over the fact that a majority of Ghanaian households remain without fire extinguishers, despite it being a legal obligation [36]. Addressing these factors through education, training, and regular drills is essential for enhancing the public's ability to respond effectively in such situations. By prioritizing education, training, and regular drills, we empower individuals with the knowledge and skills needed to respond effectively in potentially life-threatening situations. Through education, individuals gain a deep understanding of basic electrical safety measures and become aware of the potential risks associated with emergencies. This knowledge forms the foundation for a proactive and informed response. Furthermore, structured training programs provide hands-on experience, allowing individuals to practice emergency protocols in a controlled environment, building confidence and readiness [7]. Regular drills serve as vital rehearsals, reinforcing proper procedures and ensuring a smooth, coordinated response when faced with an actual electrical emergency. This multifaceted approach not only enhances public safety but also cultivates a culture of preparedness, ultimately saving lives and minimizing the impact of unforeseen electrical incidents.

**Fig. 11:**
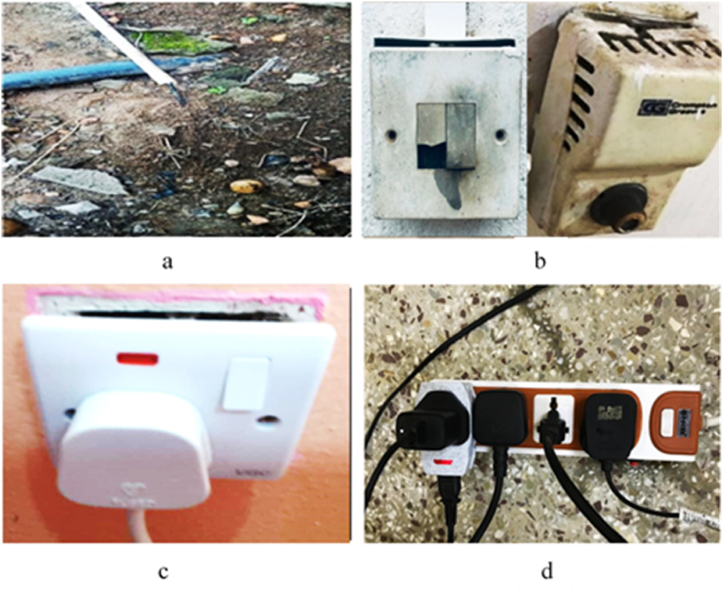
Some improper electrical connections and hazardous usage practices: (a). Bad earth connection; (b). Bad switching system; (c). Bad socket system; (d). Overloaded extension cord.

The study focused on specific electrical accidents and found that shocks (63.5 %) were the most common among respondents. An electric shock refers to the physiological reaction experienced when an individual's body comes into contact with an electrical current, typically resulting from accidental contact with live wires or electrical appliances [37]. This happens when there is direct contact with an exposed wire or a faulty electrical device, allowing the current to pass through the body. This phenomenon essentially turns the human body into a conductor of electrical energy [2]. The impact of an electric shock varies, ranging from a slight tingling feeling to potentially leading to sudden cardiac arrest. Several elements contribute to its severity, such as the level of current coursing through the body, the route it travels within the body, the duration of contact with the circuit, and the frequency of the current [37]. Overall, 73 % of the respondents reported experiencing various electrical accidents in their homes. An electrical accident can result in severe complications, including fatalities and long-term disabilities that necessitate long-term medical assistance [21]. An inspection revealed some poor electrical connections in several homes. Some of these hazards observed in the residences were documented through images, as shown in Fig. 11(a–d). These hazardous conditions pose a potential risk for accidents during the process of appliance switching and connection. A hazard is anything identified as a potential source of danger, such as exposed electrical wires, bad switches, unsafe practices, unprotected installations, overloaded socket outlets, and more, as depicted in Fig. 11(a–d). The study's findings suggest that respondents demonstrated inappropriate attitudes toward preventing home accidents.

It's critical to adhere to safe practices, such as using certified extension cables, avoiding overloading circuits, fixing exposed and opened electrical accessories, providing the correct earthing system to prevent the flow of current around exposed conductive parts, and making sure there is enough insulation, to prevent electrical accidents in homes. Maintaining safety also heavily depends on timely electrical system maintenance and routine inspections. A safe living environment also requires educating users about electrical hazards and how to handle emergencies.

Safety practices among electricity users were determined to be substandard. The majority of people are not fully aware of safety practices in the use of electricity due to a lack of proper education and information dissemination about electrical safety measures. This results in a widespread lack of knowledge about potential hazards and the correct procedures to follow during electrical emergencies. Additionally, complacency and a lack of regular safety drills contribute to this gap in awareness. Poor awareness of electrical safety practices heightens the risk of encountering invisible threats within electrical systems. This could lead to potential hazards like electrical fires, shocks, or damage to appliances [37]. Additionally, it may result in inefficient energy consumption and increased utility costs. In essence, inadequate awareness of electrical safety practices amplifies the likelihood of encountering these concealed risks, which can have serious consequences for both individuals and property [38]. Promoting electrical safety education and awareness is not just a matter of convenience; it's a cornerstone of responsible citizenship and community well-being. By instilling a strong understanding of electrical hazards and safe practices, we empower individuals to protect themselves, their families, and their properties from potential dangers. Moreover, at a broader societal level, this awareness fosters a culture of safety, reducing the incidence of electrical accidents and the associated social and economic burdens [39]. This unified approach to electrical safety education greatly enhances the overall safety and well-being of our communities. The focus group, comprised of 50 homeowners, demonstrated a substantial understanding of electrical installation inspection and testing, particularly concerning earth resistance assessment. However, there was a noticeable lack of interest and comprehension when it came to other crucial tests like insulation resistance, visual inspection, polarity, and RCD tests. This underscores a potential gap in awareness and highlights the need for further education in these critical areas of electrical safety assessment. Homeowners often show interest in the earth resistance test because it directly relates to the safety and functionality of their electrical systems. This test assesses how effectively the grounding system disperses excess electrical energy into the earth, reducing the risk of electric shock or fire hazards. Understanding the earth resistance value provides assurance that the electrical installation is properly grounded, contributing to a safer living environment [40]. Additionally, a low earth resistance value indicates a more efficient grounding system, which can enhance the overall performance and reliability of electrical equipment in the home [41], including an effective billing system.

Despite possessing this knowledge, a significant portion of homeowners have neglected the inspection of their electrical wiring for over 10 years. Furthermore, they expressed limited familiarity with CEWPs and CEWIs, and have not sought their services for the assessment or testing of their electrical systems. This indicates a gap in the proactive maintenance and safety measures concerning their electrical installations. As time passes and electrical systems are utilized, they naturally undergo wear and tear. Therefore, it's imperative to conduct regular inspections and tests to guarantee their ongoing safety and functionality. These routine safety evaluations are commonly referred to as periodic inspections and testing [20]. As per the guidelines provided by the Energy Commission of Ghana, it is recommended that owner-occupied homes undergo electrical inspection and testing every 10 years, while rented homes should have this done every 5 years [42]. This directive is in accordance with the wiring regulations of 2011, which was officially ratified by parliament on February 24th, 2012. These regulations serve as a framework to govern professionals involved in electrical wiring and installation tasks. They are designed to uphold the enforcement of minimal standards for electrical wiring within facilities, ensuring the safety of individuals, animals, and assets from potential threats related to the distribution and use of electrical energy. According to these regulations, only CEWPs and CEWIs, per the regulations, will have the legal authorization to conduct indoor electrical wiring and inspections [14].

Facility owners bear the responsibility of ensuring the safety and integrity of their electrical systems. As of the end of 2016, it was mandated that all facility owners conduct a comprehensive inspection and testing of their electrical wiring. This process was designed to ascertain that the wiring met the requisite standards and posed no hazards. Following the examination, facility owners were required to obtain an Inspection and Testing Certificate from a CEWI accredited by the Energy Commission. This certificate served as a testament to the electrical system's compliance with established safety and quality guidelines [14]. However, it's worth noting that none of the homeowners possessed this certificate at the time of the research. Further investigations indicated that only buildings less than 5 years old had this certificate, primarily because they involved CEWP or CEWI in their electrical wiring projects. An installation carried out by unqualified and unlicensed electricians, without adherence to IEEE regulations, poses a significant risk [20].

In interviews and discussions with the group, they revealed various reasons for their diverse hesitation to test their electrical installations:

“The electrical system hasn't experienced any noticeable issues, and assume that it's functioning fine, leading them to overlook the need for testing. Many homeowners have not fully grasped the importance of regular electrical testing. They are apprehensive about the potential costs associated with testing, especially if they believe their system is in good condition. Additionally, coordinating with a certified inspector, arranging for the testing, and potentially addressing any issues can be viewed as an inconvenience.”

It should be noted that this initiative by the Energy Commission aimed to enhance electrical safety standards across various facilities, mitigating potential risks associated with faulty wiring. It reinforced the imperative for facility owners to prioritize regular assessments of their electrical infrastructure to guarantee the well-being of occupants and protect valuable assets. In addition to ensuring the safety of your family, regular testing and inspections can also lead to cost savings. By conducting routine tests, potential issues and invisible threats can be identified early, allowing for planned electrical repairs. This minimizes or even eliminates disruptions to your day-to-day operations. Being proactive in addressing these concerns enables you to anticipate repair costs, ultimately reducing any potential revenue loss and minimizing disruptions to your regular activities.

Hence, it is strongly advised that the commission take a proactive stance in enforcing this crucial safety guideline for homeowners nationwide. This can be achieved through a sustained televised education campaign, supplemented by routine home inspections to ensure compliance and issuance of Inspection and Testing Certificates by CEWIs. This comprehensive approach will help safeguard lives and property from potential electrical hazards.

Ultimately, the study delved into the comprehensive assessment of safety provisions within residential buildings. Respondents expressed a degree of uncertainty concerning the safety measures implemented in their households with electricity usage. This uncertainty could be attributed to a limited awareness regarding safety practices, as well as the correct procedures to follow in the event of an electrical emergency while using electricity. The research uncovered various electrical wiring challenges faced by consumers, ranging from issues with earthing systems to faulty wiring, all of which posed significant risks like electrical shock, fire outbreaks, and increased electricity bills due to leakage currents. While some households had robust earthing systems in place, only a few had both earthing systems and RCDs installed. Notably, around 10 % of the respondents were found to have subpar safety system installations, a trend predominantly observed in older buildings.

The IPMA and NCA analysis provide valuable insights into the relationship between awareness of electrical hazards, awareness of electrical safety practices, and awareness of proper safety procedures on overall electrical safety. Each chart demonstrates the influence of these awareness factors on overall safety in electrical usage. Both analyses indicate that to achieve optimal safety outcomes, efforts should be focused on enhancing awareness of electrical safety practices and electrical hazards. These factors not only demonstrate high importance and moderate to significant performance but also significantly influence the ceiling of achievable safety levels. While maintaining high performance in awareness of proper safety procedures remains necessary, prioritizing interventions in the more critical areas identified by both IPMA and NCA, namely, awareness of electrical hazards and electrical safety practices, will lead to substantial improvements in overall electrical safety.

While new buildings must adhere to established electrical safety regulations, the concern lies with older structures, which present a greater challenge in terms of electrical safety. Given that electrical hazards can lead to severe injuries or even fatalities, prioritizing safety measures becomes paramount in any environmental setup [19].

These findings underscore the vital importance of disseminating comprehensive information on electrical safety and awareness to electricity users, a responsibility that lies with authorized regulatory bodies in Ghana. This perspective aligns with the belief of [43], who argue that enhancing knowledge, skills, attitudes, and habits through proper safety education is crucial in preventing domestic accidents. It's clear that information is invaluable in ensuring individual safety and promoting heightened awareness in this regard.

### 5.2 Study implications

This lack of awareness of several critical risks and safety practices constitutes a major safety risk. Improper electrical appliance and wiring handling can result in devastating electrical fires and tragedies. Lives are at risk, and it is critical to arm ourselves with the knowledge we need to avoid such catastrophes. Aside from safety problems, we see severe property damage as a result of this misinformation. Overloaded circuits and improper wiring are all too typical when people do not understand electrical systems. The effects can be disastrous, resulting in costly repairs and financial strain on families.

Personal well-being is also jeopardized. Electric shocks, burns, and even fatalities can result from inappropriate electrical equipment use. Exposed wiring and defective equipment become potential hazards, and we leave ourselves open to these dangers if we do not have the proper expertise. Furthermore, the financial cost of a lack of electrical expertise cannot be overstated. Higher utility expenses occur from inefficient energy use caused by inappropriate use of equipment and appliances. This not only has an impact on household budgets but also places an unneeded load on our electricity grid.

Another area where little understanding can have major consequences is compliance with local electrical codes. Failure to follow these requirements may result in fines and penalties, putting another financial strain on families already trying to make ends meet. Taking into account the impact on the lifespan of our equipment, improper voltage or power supply emanating from poor installation work and non-compliance with local electrical codes might cause premature failure, needing frequent replacements. This not only affects our finances but also contributes to environmental waste. In the face of these issues, we must emphasize the necessity of electrical safety education and awareness. Encouragement of responsible electrical use, knowledge of grounding and surge protection, and promotion of the use of safety devices are all critical measures in reducing these practical consequences.

The findings from the IPMA and NCA ceiling line charts underscore the importance of targeted educational interventions aimed at increasing awareness of proper safety procedures. Policymakers and electrical safety advocates should prioritize campaigns and training programs that focus on these areas to maximize their impact. Additionally, the plateau effect observed in the awareness of electrical safety practices suggests that once a certain level of awareness is achieved, additional factors such as regular inspections, enforcement of regulations, and availability of safety devices might be necessary to further enhance safety.

The analysis highlights the critical role of awareness in promoting electrical safety. Both general electrical safety practices and specific safety procedures are essential, but the latter has a more significant impact on overall safety outcomes. These insights should inform future strategies and policies aimed at reducing electrical hazards and improving safety in residential settings. By addressing these key areas, it is possible to create safer environments and prevent the adverse consequences associated with electrical mishandling.

Ultimately, in emergencies, the consequences of poor knowledge can be magnified. The ability to take swift, informed action during power outages or electrical malfunctions can mean the difference between safety and further peril. It is our collective responsibility to address this issue head-on, to empower our communities with the knowledge they need to create safe, efficient, and sustainable living environments by raising awareness and promoting education on electrical safety practices to ensure the well-being of families and the resilience of the communities in the country.

## 6 Conclusion and recommendation

The research delved into the safety standards of residential structures and the level of awareness among electricity users in Sokode–Etoe, situated in the Ho Municipality of the Volta Region, Ghana. The findings highlight a significant lack of awareness among energy consumers regarding electrical safety practices and proper procedures in the event of an electrical emergency. This deficiency is reflected in the notable incidence of electrical mishaps witnessed in households. The study found that being male was associated with a higher likelihood of knowing the correct procedures for handling an electrical emergency; (2.543; 95 % C·I.: 1.302–4.968 (*p-value* = 0.006 < 0.05)). Furthermore, the mean score for the overall level of safety in the electricity usage test demonstrated a statistically significant deviation of 1.96 (95 % CI: 1.83–2.08) from the standard score of 5.0 [t (199) = −30.400, p-value = 0.000 < 0.05], indicating considerable divergence. Respondents expressed uncertainty regarding the overall safety level associated with electricity utilization within their households. A majority of homeowners have never engaged a certified electrical inspector for inspection or testing in the past decade. Moreover, many are unsure about the overall safety protocols in place within their households concerning electrical usage. The results from the IPMA and NCA analyses underscore the critical role of various awareness factors in enhancing overall electrical safety.

As per the research's findings, it's imperative to enhance the electrical safety knowledge of residential consumers through diverse and innovative awareness strategies. For instance:

Government bodies, non-governmental organizations, electrical providers, and appliance manufacturers should collaborate to educate electricity users about potential hazards. This education campaign could leverage various communication channels such as television, radio, posters, public seminars, the Internet, and other platforms.

Organize interactive workshops and live demonstrations within community settings, educational institutions, and workplaces to illustrate correct safety protocols and methodologies. These dynamic sessions enable participants to directly interact with the safety apparatus and pose inquiries at the moment.

Partner with social media influencers, bloggers, and renowned personalities boasting substantial followings to support and promote electrical safety messages, thus broadening the outreach to diverse audiences. Genuine endorsements from respected figures can bolster the credibility and effectiveness of the educational campaign.

Develop customised mobile applications that offer interactive courses, quizzes, and safety checklists focused on electrical safety. Additionally, these applications may incorporate functionalities like augmented reality to promptly detect potential dangers.

Launch targeted social media campaigns on platforms like Instagram, TikTok, and Twitter to raise awareness about electrical hazards and safety practices. Utilize engaging content formats such as short videos, infographics, and user-generated challenges to capture attention and encourage sharing.

The adoption of new technologies plays a significant role in enhancing electrical safety. The integration of Internet of Things (IoT) devices in home electrical systems offers real-time monitoring and control, further improving safety and efficiency.

All electrical installations undergo inspection and testing every 5–10 years in adherence to IEEE regulations and the Energy Commission of Ghana's guidelines. This proactive approach allows for the early detection of potential issues and hidden threats, enabling planned repairs.

Electricity supply authorities should conduct regular inspections at locations where electricity is utilized to identify abnormalities like unauthorized connections. It's worth noting that during inspections, it was observed that some houses with inadequate earthing and protective measures were obtaining electricity from their neighbours, which was not officially sanctioned.

In any environmental context, prioritizing safety is paramount due to the significant risk of harm or fatality posed by electrical hazards to individuals. To prevent power-related accidents in the township and municipality, a collective effort is imperative to raise awareness about the risks and safe practices associated with electricity. Recognizing the importance of electricity in our lives, it is crucial to provide education on its safe and effective use to meet our daily needs. This is essential to mitigate the significant risks of death and injuries associated with electrical hazards.

## 7 Limitation of the study

This study's scope is confined to the Sokode-Etoe township. However, the insights gained from this survey hold substantial value given the scarcity of research on electrical hazards and safety practices in this region, as well as in Ghana at large. Future research endeavours could broaden their scope to include the Ho municipality and other municipalities across Ghana. Additionally, conducting extensive, prolonged surveys on broader and more diverse samples, while expanding the analysis to cover various categories of hazard and safety knowledge, would be instrumental.

Moreover, several pioneering research proposals aimed at examining electrical hazards and safety protocols among residential electricity users have been suggested. These include cross-cultural studies intended to elucidate variances in electrical safety practices and perceptions across diverse demographic groups and geographic regions in Ghana. Additionally, there is the proposition of leveraging machine learning algorithms to scrutinize datasets encompassing electrical consumption patterns, household attributes, and historical incidents, intending to prognosticate and preempt prospective electrical hazards. These endeavours would not only pinpoint additional gaps in health and safety management but also contribute to the development of comprehensive awareness campaigns aimed at enhancing sustainable safety management strategies in the country.

## Consent to participate

Written informed consent was obtained from all individual participants included in the study.

## Ethics statement

Approval for this study was granted by the ethics committee of Ho Technical University (HTU/DRI/EC2023-019). Prior to conducting face-to-face questionnaire interviews and inspections written informed consent was obtained from all participants, including homeowners and tenants.

## Data availability statement

The data associated with the study will be made available upon reasonable request.

## Funding

The authors received no direct funding for this research.

## CRediT authorship contribution statement

**Frank Kulor:** Writing – review & editing, Supervision, Project administration, Methodology, Conceptualization. **Michael W. Apprey:** Writing – original draft, Validation, Methodology, Formal analysis, Data curation, Conceptualization. **Kafui T. Agbevanu:** Writing – review & editing. **Gabriel K. Gasper:** Writing – review & editing. **Judith A. Akorta:** Writing – review & editing.

## Declaration of competing interest

The authors declare that they have no known competing financial interests or personal relationships that could have appeared to influence the work reported in this paper.

## Abbreviations

Sigf: Significance
OR: Odds Ratio
C. I.: Confidence Interval
Exp(B): Exponentiated Coefficient
df: Degree of freedom
n/N: Total number of respondents
GNFS: Ghana National Fire Service
RCD: Residual Current Device
FG: Focus Group
SPSS: Statistical Package for Social Sciences
JHS: Junior high school
SHS: Senior high school
CEWP: Certified Electrical Wiring Professional
CEWI: Certified Electrical Wiring Inspector
L.I.: Legislative Instrument
IEEE: Institute of Electrical and Electronic Engineers
Hz: Hertz
NCA: Necessary Condition Analysis
IPMA: Importance-Performance Map Analysis
CE-FDH: Ceiling Envelopment - Free Disposal Hull
CR-FDH: Ceiling Regression - Free Disposal Hull
PLS-SEM: Partial Least Squares Structural Equation Modelling
