# Supplementary material for: Invisible threats: An investigation of electrical hazards and safety practices among residential electricity consumers
Source: Heliyon. 2024 Jul 11;10(14):e34470. doi: 10.1016/j.heliyon.2024.e34470 (PMC11304026; doi:10.1016/j.heliyon.2024.e34470)
Supplement: Multimedia component 1 [file mmc1.docx]

**
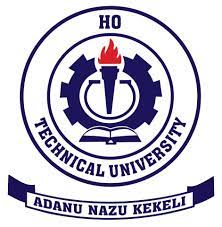
HO TECHNICAL UNIVERSITY**

**FACULTY OF ENGINEERING**

**DEPARTMENT OF ELECTRICAL AND ELECTRONIC ENGINEERING**

**QUESTIONNAIRE FOR THE SURVEY ON “INVISIBLE THREATS: AN INVESTIGATION OF ELECTRICAL HAZARDS AND SAFETY PRACTICES AMONG RESIDENTIAL ELECTRICITY USERS”**

Dear Respondent,

Thank you for taking part in our survey, designed to gain insights into awareness of electrical hazards and safety practices among residential electricity users. Your input will enhance our understanding of the current situation and help promote safer electrical practices. Please be assured that all information collected will be treated with utmost confidentiality and solely used for academic purpose. Access to the data will be restricted to the lead investigator and the data collector. Your cooperation is greatly appreciated.

Sincerely Yours

Please answer the following questions honestly and to the best of your knowledge.

**Section A: Respondent’s Details (Please tick [√] only one box)**

1. **Gender**: i. Male ii. Female
2. **Age:** i. 20-25 ii. 26-45 iii. 41-60 iv. >60
3. **Educational Qualification:** i. None ii. JHS iii. SHS iv. Tertiary
4. **House Status:** i. Owner ii. Tenant

**Section B: Awareness of electrical hazards, proper safety procedures and accidents**

1. How would you rate your general awareness of potential hazards associated with the use of electrical appliances and equipment?

∎Please tick **[√] only one box** in each row.

| **S/No** | **Items** | **Aware** | **Unaware** |
| --- | --- | --- | --- |
| 1. | Coiling extension leads on the drum. |  |  |
| 2. | Covering ventilation holes in electrical equipment. |  |  |
| 3. | Use of high lighting fitting. |  |  |
| 4. | Removing the plug from an electrical socket by tugging on the cord. |  |  |
| 5. | Overloading of extension cords. |  |  |
| 6. | Use of damaged outlets and switches. |  |  |
| 7. | Use of systems with exposed wires. |  |  |

1. How would you rate the awareness of proper safety procedures to follow in the event of an electrical emergency (e.g., short circuit, electrical fire)?

i. Aware ii. Unaware

1. Please indicate one (1) major electrical fatality encountered during the use of electricity.

Please tick **[√] one box only** as your **MAIN** electrical fatality encountered.

| **S/No** | **Electrical Accident** | |
| --- | --- | --- |
| 1. | Burns |  |
| 2. | Electrical shock |  |
| 3. | Electrocution |  |
| 4. | Fire |  |
| 5. | Indirect (fall) |  |
| 6. | None of the above |  |

**Section C: Awareness of electrical safety practices**

1. How would you rate your general awareness of electrical safety practices associated with the use of electrical appliances and equipment?

∎Please tick **[√] one box only** in each row.

| **S/No** | **Items** | **Aware** | **Unaware** |
| --- | --- | --- | --- |
| 1. | The extension cord is fully uncoiled from the drum. |  |  |
| 2. | Unplug unused devices. |  |  |
| 3. | Avoid plugging the heater on an extension cord. |  |  |
| 4. | Avoid touching power equipment with a wet body. |  |  |
| 5. | Conduct regular inspections. |  |  |
| 6. | Avoid overloading circuits. |  |  |

**Section D: Inspection and testing of electrical installation** *(For home owners only)*

1. Which of the following electrical testing methods are you familiar with?

Please tick **[√] just one box only** as your **MAJOR** knowledge in electrical testing method

| **S/No** | **Electrical tests** | |
| --- | --- | --- |
| 1. | Visual inspection |  |
| 2. | Earth resistance test |  |
| 3. | Insulation resistance test |  |
| 4. | Polarity test |  |
| 5. | RCD test |  |
| 6. | All of the above |  |

1. Have you inspected and tested your building over the last ten (10) years by engaging certified electrical wiring professional and inspector?
2. Yes ii. No

**Section E:** **Overall level of safety in the use of electricity**

1. To what extent do you agree with the following statement indicated in the table below? Please tick **[√] one box only** with the following meaning;

**1 = Strongly Disagree; 2 = Disagree; 3 = Uncertain; 4 = Agree; 5 = Strongly Agree**

| **S/No** | **Item** | **1** | **2** | **3** | **4** | **5** |
| --- | --- | --- | --- | --- | --- | --- |
| 1. | Overall, I am convinced that based on how i/we use electricity, the safety of my/our household is at risk. |  |  |  |  |  |

**Thank you for taking the time to complete this survey. Your input is valuable in promoting electrical safety and preventing hazards at home.**
